# Revival of positive nostalgic music during the first Covid-19 lockdown in the UK: evidence from Spotify streaming data

**DOI:** 10.1057/s41599-023-01614-0

**Published:** 2023-03-29

**Authors:** Timothy Yu-Cheong Yeung

**Affiliations:** 1 Centre for European Policy Studies, Brussels, Belgium; 2 KU Leuven, Leuven, Belgium

**Keywords:** Cultural and media studies, Psychology

## Abstract

This work shows that positive old music listening surged during the early phase of the Covid-19 pandemic, suggesting a rise in preference for nostalgia and positivity in music. Using the music streaming data of Spotify users in the UK and employing multivariate regression analysis, this work documents that users were more likely to listen to songs older than 5 years during the national lockdown that began in late March 2020 compared with the pre-lockdown period. Such a change in preference was not observed in the same period in 2019. Meanwhile, more frequent listening to old music is found in samples of positive songs and also negative songs. This suggests that the preference for nostalgic music is to a certain extent independent of the positivity bias during the pandemic found in the literature. Yet, this work also provides evidence that the nostalgia-seeking behaviour and the preference for positive songs reinforced each other during the lockdown as the surge in positive old music was more persistent than that in positive recent music.

## Introduction

The Covid-19 pandemic is unprecedented in modern times. During the first wave in 2020, lockdown or confinement measures entailed drastic changes for many people. The measures were all-inclusive, affecting all of the population at the same time on an extraordinary scale. The lockdown, which abruptly stopped people from moving around and banned customary social and economic activities, was impactful on behaviour and emotions. The first lockdown to halt the spread of Covid-19 in 2020 was especially impactful on behaviour and emotions, as most people had no experience of being ordered to stay at home for an unknown length of time. The degree of social isolation caused by lockdowns was exceptional, with adverse effects on mental health (Banks and Xu, 2020; Elmer et al., 2020; Panchal et al., 2021; Pieh et al., 2021) and impacts on human behaviours.

During a difficult time, people adopt emotion regulation strategies to combat negative feelings (Restubog et al., 2020), and even avoidance can produce some short-term positive effects (Panayiotou et al., 2021). Listening to music is one of the emotion regulation strategies used in ordinary times (Thoma et al., 2012) and during the pandemic (Howlin and Hansen, 2022). Previous research has shown that music significantly affects one’s mental condition (Thoma et al., 2012; Swaminathan and Schellenberg, 2015; MacDonald et al., 2013).

The pandemic sparked off a new line of research about music during Covid-19 (Howlin and Hansen, 2022). It has been shown that listening to music was a very effective strategy for emotion regulation to cope with the negativity induced by the pandemic (Fink et al., 2021; Gibbs and Egermann, 2021; Granot et al., 2021; Martínez-Castilla et al., 2021; Martín et al., 2021; Mas-Herrero et al., 2020).

A subsequent question concerns whether there were any changes in music listening behaviour in response to the pandemic. As music listening is an emotion regulation strategy, the changes in circumstances would have very likely altered listeners’ music choices. Most researchers have attempted to identify any changes through surveys. Ferreri et al. (2021) found that respondents sought new and happy songs as well as music from childhood during the pandemic. Fink et al. (2021) found that self-reported listening to nostalgic music increased by 36%, while Granot et al. (2021) found that nostalgic music listening was associated with distress.

As surveys rely on self-reported evidence and pre-pandemic benchmarks may be lacking, this work attempts to supplement the new line of research by leveraging the abundance of music streaming data. The study collects data on the music streaming choices of Spotify users and examines behavioural changes in nostalgic and positive music listening during the early phase of the pandemic.

Indeed, the literature has given a probable explaination. The literature shows that negative emotions predict nostalgia (Wildschut et al., 2006), that nostalgia leads to some positive effects (Van Tilburg et al., 2013; Cheung et al., 2017) and that music can evoke nostalgia (Barret et al., 2010). Scholars found that music nostalgia was an emotion regulation strategy used to combat the negative impacts of the pandemic (Gibbs and Egermann, 2021). These authors found that nostalgic music was defined personally for a reason, such as its relation to memories of specific events, places, people or personal identity.

Using multivariate cross-sectional and also time series regression analyses, this work provides evidence of increases in the frequency of listening to both nostalgic and positive songs during the first Covid-19 lockdown in the UK. First, the paper finds that the first lockdown in the UK significantly altered music listening behaviour: people tended to listen to the music of the past. This finding passes two robustness tests. First, I verify that the lockdown triggered a change in old music listening behaviour by checking the change in the behavioural trend around the lockdown starting date (26 March 2020, *t* = 0). I find that the change in trend is most evident at *t* = −1, suggesting that there was indeed a behavioural change roughly around the lockdown starting date. Second, I verify if the pattern is just an annual one that could also be found in the data for 2019. I perform a similar check and find no upward change in the trend of old music listening behaviour from mid-March to early April 2019, showing that the pattern in 2020 is particular and very likely associated with the 2020 lockdown.

The study moves on to look at some of the more prominent ‘revived’ songs and what types of songs they are. A brief assessment of their lyrics points to the result that they are mostly positive songs. Using the same streaming data, I conduct a time series analysis that suggests a few interesting conclusions:

- Listening to old music is more frequent after the lockdown in both the sample of positive songs and the sample of negative songs.
- Listening to old music in the sample of positive songs follows an upward trend even beyond the initial incremental increase, while this continuous increase is absent in the sample of negative songs.
- The average positivity level of the sample of recent songs increases at the beginning of the lockdown but wanes as time goes on (reversal).
- The average positivity level of the sample of old songs increases gradually after the lockdown without a significant reversal during the sample period.

## Materials

Spotify publishes a country’s daily top 200 songs (ranked by the number of plays) and provides an application programming interface for developers and researchers to obtain data on the songs. Data from approximately 4 trillion plays of more than 4000 unique charted songs streamed in the UK from 1 January to 31 July 2020 are studied in this work. Yeung (2020) has studied music streaming on Spotify for the same period in six European countries including the UK. This work focuses on the UK and provides a deep dive into the revived songs.^1^ The UK is chosen for two main reasons. First, the UK is the top country among the six included in Yeung (2020) in terms of Spotify’s music streaming volume. Second, the dominance of English songs in the UK allows for a less scientific but more intuitive analysis of their lyrics, which serves as a prelude for a more scientific study of their valence levels.

The sample period covers the whole of the first wave and is the best timeframe for studying the instant behavioural changes due to the lockdown, since people were much more prepared and informed during the subsequent waves. The variables obtained include the song titles, their daily number of plays, their release dates and their audio features provided by Spotify. Lockdown dates are taken from official announcements^2^ and Covid-19 incidence rates are collected from the EU Open Data Portal.^3^

## Methods

The first challenge of this research is to operationalise the concept of nostalgic music. ‘Nostalgic’ is a relative adjective. Nostalgic music to people in their 30s today may refer to songs of the 1990s, while adolescents may regard albums of their favourite singers from 5 years ago as nostalgic. It is thus difficult to pin down a universal definition of a choice being nostalgic.

If we could identify Spotify users and their ages, we might be able to specify a flexible time frame for constructing a relative definition. As Spotify does not make the personal information of users publicly available, this work instead relies on an absolute definition of ‘old’ music, which is supposed to induce nostalgia. Any songs older than 5 years on the day of being played are considered old music, in contrast to recent music. A binary measure of old music is constructed accordingly.

Setting the threshold at 5 years is arguably reasonable given that, first, to evoke nostalgia a song must be familiar to the listener, i.e. a song from one’s past; and second, more than half the user base is aged under 34.^4^ Understandably, some readers may not agree with describing old songs as nostalgic; old songs do not necessarily give rise to listeners’ nostalgia. Stepping back, I ask, if a lockdown induced more frequent listening to old songs, what is a reasonable explanation? The most straightforward one is that music listeners sought nostalgia, which old songs may provide.

Notably, nostalgic songs are not necessarily songs *released* during one’s past, but songs one *listened* to in the past. Meanwhile, only old songs can satisfy one’s demand for music nostalgia. Was there such a demand? The existing literature points to similar trends in the proliferation of nostalgic entertainment or nostalgia-seeking behaviour during the pandemic (Gammon and Ramshaw, 2021; Gibbs and Egermann, 2021; Howlin and Hansen, 2022).

Discarding this explanation, one does not identify many others. One possible explanation, which this paper will test, is that the revival of old songs is a result of a need for positivity. If old songs are on average more positive, the need for positivity would lead to a surge in listening to old music. Or simply, old songs are better songs, which were somehow discovered by Spotify users.^5^ Similar explanations that do not regard nostalgia as a driving force would need to identify the possible channels through which music listeners know those old songs. Even if one identifies a possible channel, it would need to show that the channel impacted society so much that it altered the general trend observed in Spotify streaming data. To remain prudent, before reaching the end of the empirical analysis this work resorts to the division of music into old and recent songs, but then apply the explanation this work suggests and infer whether people listened more frequently to nostalgic music during the early phase of the pandemic.

The data, however, do not allow us to infer the emotional status of the music listeners, or pinpoint the motive behind any behavioural changes. The objective of this study is to leverage the observational data on a popular music streaming platform and draw insights for the understanding of human behaviour during the pandemic.

**Fig. 1:**
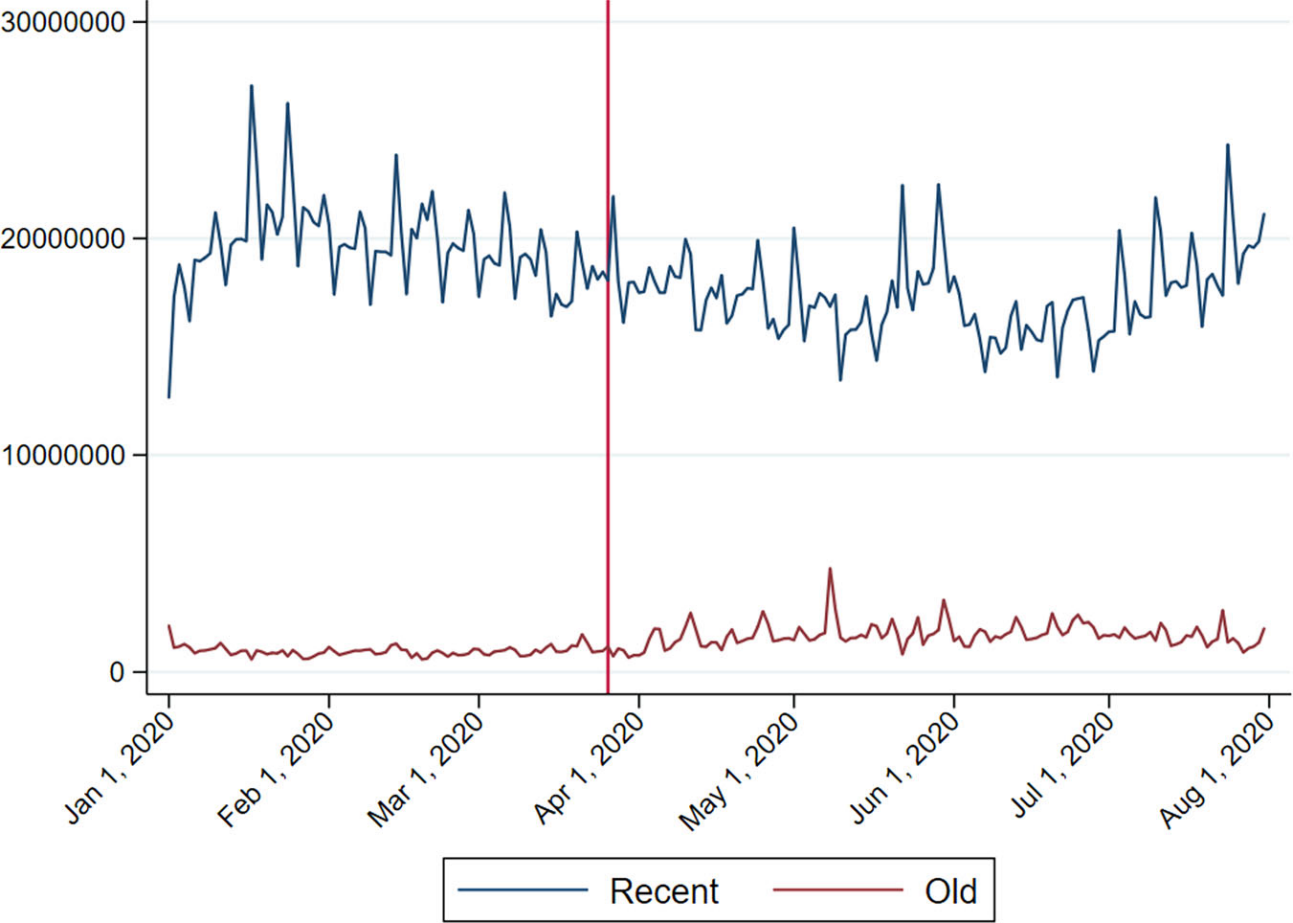
UK daily number of plays (top 200 only), January–July 2020. *Note*: The vertical reference line indicates the beginning of the lockdown on 26 March 2020. *Source*: Spotify.

**Fig. 2:**
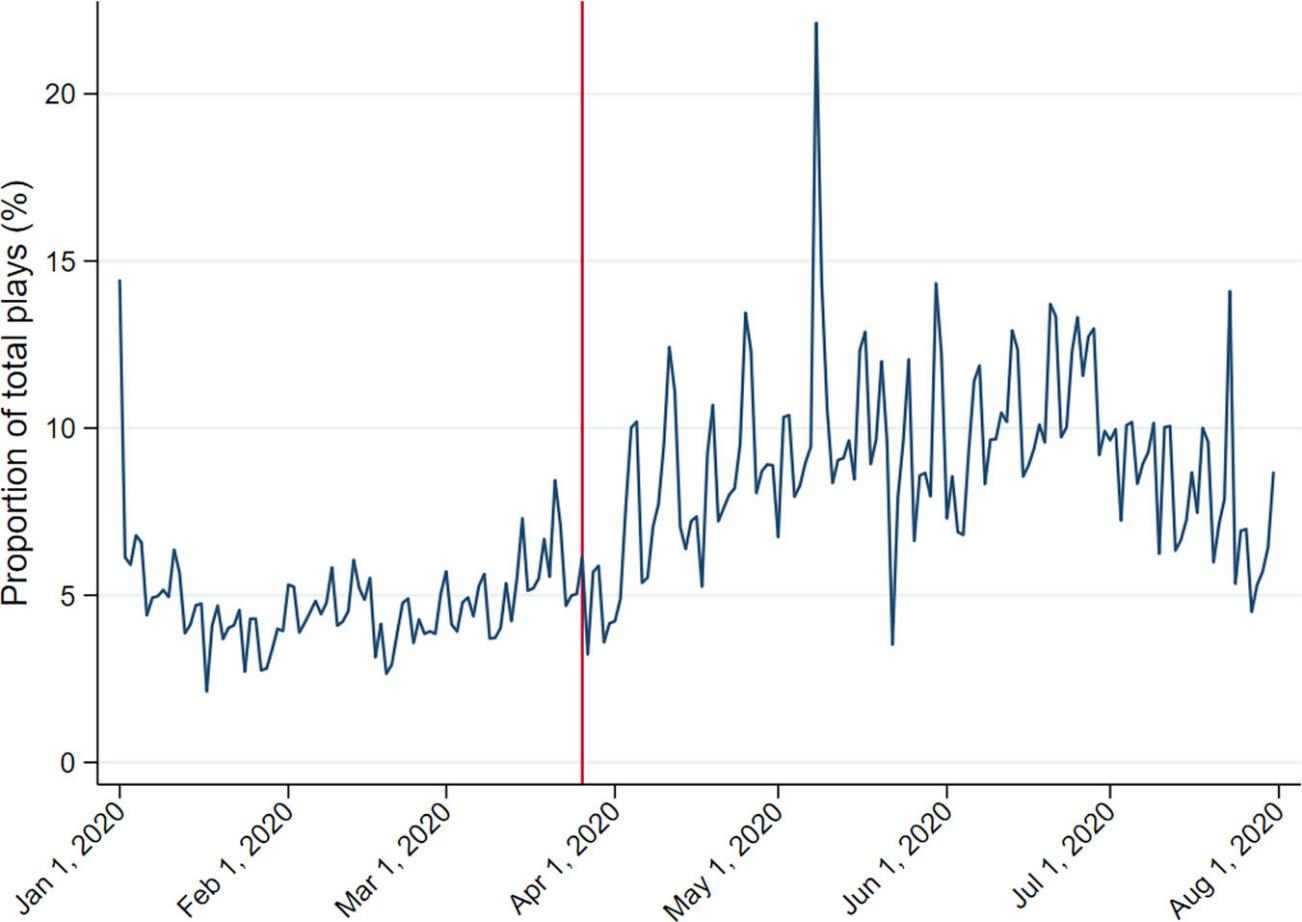
UK daily plays expressed as a proportion of all plays of top 200 songs, January–July 2020. *Note*: The vertical reference line indicates the beginning of lockdown on 26 March 2020. *Source*: Spotify.

Figure 1 shows the number of plays of old and recent songs from January 2020 to July 2020 with a vertical reference line indicating the beginning of the lockdown on 26 March.^6^ Although old music only accounts for a relatively small number of plays, the upward trend since the end of March is fairly distinguishable. Figure 2 gives a clear illustration by presenting the information as a proportion of the total number of plays of daily top 200 songs and concentrates on the region below 25%. The rise in old music is easily recognisable.

To explain the choice of music, I employ a logistic regression in which each play is a choice between a set of old songs (released more than 1825 days before the day of observation, *t*) and a set of recent songs. Each charted song on a day is associated with a binary variable indicating its old/recent dimension (old music listening = 1), which is the dependent variable and weighted by its frequency of plays during the day in the logistic regression.^7^ Note that the dataset is truncated as the identity and number of plays of the songs ranked below 200 are not publicly available. This problem is not prohibitive since the number of decisions being considered in the regression is large, reaching roughly 20 million a day (more than 4 trillion in the whole sample period) in the UK.

**Fig. 3:**
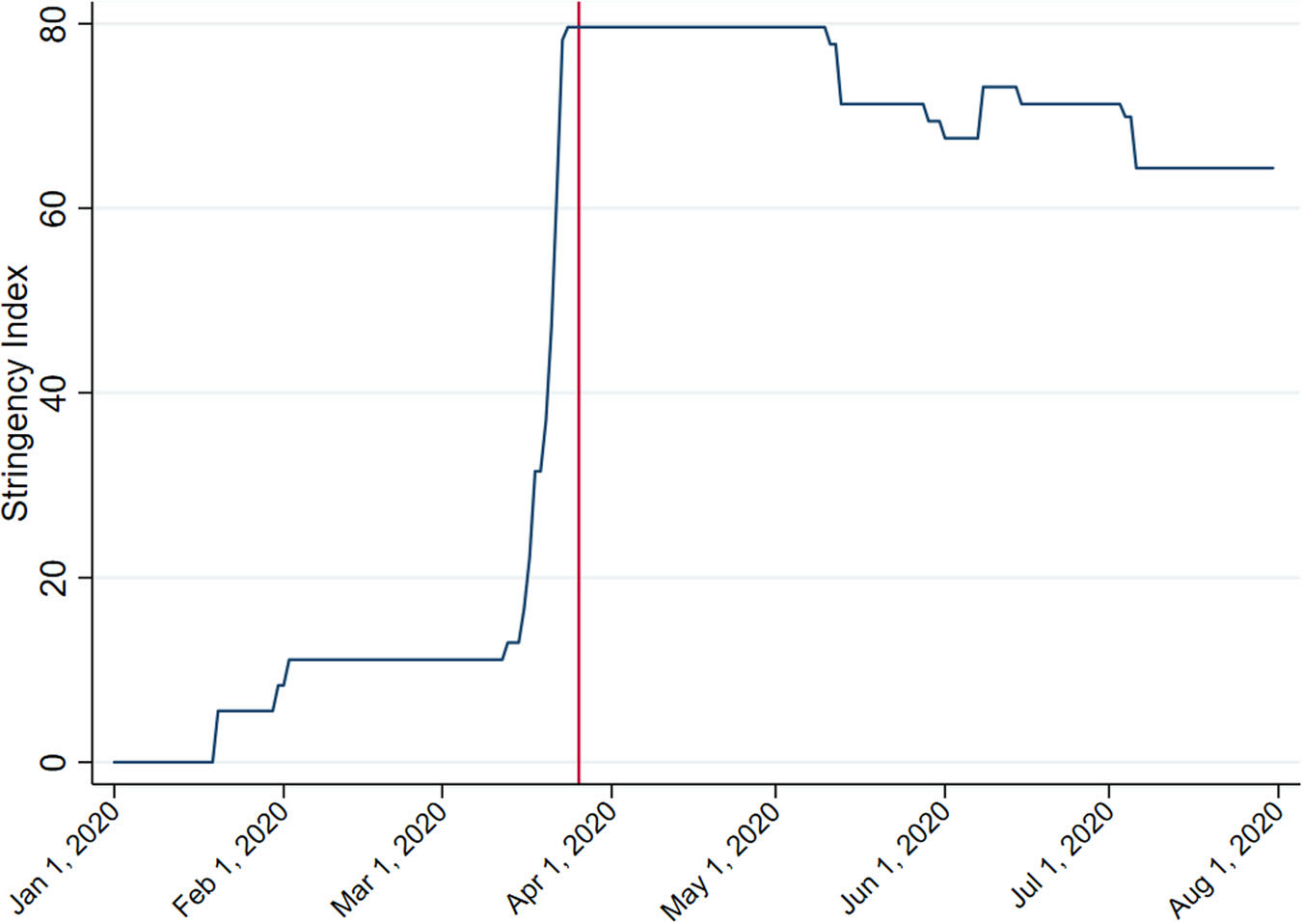
Oxford Stringency Index (UK), January–August 2020. *Source*: COVID-19 Government Response Tracker (Hale et al., 2021).

The main explanatory variable is a lockdown indicator (equals 1 if *t* ≥ *t*^L^, the lockdown implementation day, and 0 otherwise). No ending date of the lockdown is coded for two reasons. The first is that the lockdown was gradually relaxed, with some restrictions remaining throughout the summer. The second is that the lockdown was announced only shortly before its actual implementation, as the government had wanted to minimise chaotic traffic as much as possible. By contrast, the relaxation was announced some weeks before it took place. One would therefore expect the lockdown to have induced a shock, but the relaxation would have led to only gradual adjustments. As the model allows for a quadratic trend in old music listening during the in-lockdown period, it should be sufficient to capture a possible non-linear effect from the beginning of the lockdown to its progressive relaxation in July. To support the inclusion of a non-linear trend, I check with the Oxford Stringency Index of the UK and identify a sharp increase in stringent measures in late March, followed by a gradual relaxation from the end of April, as shown in Fig. 3.

Another explanatory variable of interest is the Covid-19 incidence rate, which enters the regression as the natural logarithmic transformation of the 7-day moving average of the number of daily confirmed Covid-19 cases per million of the population. The 7-day moving average is computed by averaging the numbers of new Covid-19 cases per million over the past 6 days and the day of observation. This work employs an equal-weighted 7-day moving average to measure the extent of Covid-19 infections because it gives a better measure of how people perceived the pandemic than the daily number. It also corrects the reporting delays of weekends, holidays and some exceptional negative values (ex-post adjustments). The aim of the regression analysis is to determine whether the lockdown caused users’ music listening behaviour to change and to check if, on top of the lockdown effect, the extent of Covid-19 infections provides additional explanatory power to the model.

To verify that the finding is sufficiently robust, I carry out two additional checks. If the lockdown induced a change in behaviour, the most pronounced change in trend should be observed around the actual lockdown day. Regressions with 20 other hypothetical dates, ranging from 10 days before to 10 days after the first lockdown day, are run to see if we can credibly infer causality from the lockdown to the behavioural change.

Another robustness check is to regress the same model but shift the window of analysis to the same period (January–July) in 2019. This check ensures that the change in preference around mid-March is not an annual pattern. It could be possible that, for some reason, the tendency to listen to old music takes a distinct turn and goes up every year in mid-March and then falls towards summer.^8^ Again, I examine whether the trend changes markedly at a hypothetical break. A no-result from this check would be a strong piece of evidence that the rise in old music preference is not an annual pattern. That would support the hypothesis that the lockdown was a significant factor driving nostalgic music listening behaviour in the first Covid-19 lockdown in the UK.

The second part of the paper aims to determine whether the rise in listening to old music is only a consequence of more frequent listening to positive music. This challenge is reasonable, as a short study of the lyrics of some of the more prominent revived songs shows that they are on average very positive songs. To find out if nostalgia-seeking is the most plausible explanation, I construct four variables, namely, the net numbers of plays of old positive, old negative, recent positive and recent negative songs, and then conduct a time series analysis to check if the lockdown significantly altered the trends of these four variables.

## Regression analysis

The empirical analysis relies on logistic regression with standard errors clustered in days. The analysis employs a difference-in-difference approach, where lockdown is considered a treatment and the period since the lockdown is the treated sample. The focus of the analysis is whether the lockdown led to a change in old music listening behaviour, revealed by a change in the trend. The probability of an event *Y* = 1 (an old song is chosen) is denoted by *p*. The log-odds is thus:

1 $${\rm {logit}}\left( p \right) = {\rm {ln}}\left( {\frac{p}{{1 - p}}} \right)$$

I assume that the log-odds of *Y* = 1 is explained by a set of explanatory variables that includes a lockdown indicator and its interaction with a quadratic trend and the 7-day moving average of the Covid-19 incidence rate. The log-odds is modelled as the following:

2 $$\begin{array}{l}{\rm {logit}}\left( p \right) = \alpha _1 + \alpha _2{\rm {Lockdown}}_t + \beta _1t + \beta _2t^2 + \\ \beta _3{\rm {Lockdown}}_tt + \beta _4{\rm {Lockdown}}_tt^2 + \beta _5{\rm {Covid}}_t + x^\prime \gamma \end{array}$$

where the vector ***x*** of explanatory variables includes the number of newly released songs, the log of the total number of plays of the day, the average oldness level of the same day in 2019 and the fixed effects of the day of the week (Monday, Tuesday and so on). For easier interpretation of the result, I centred the time variable *t* on the first lockdown day. Newly released songs are those released within 30 days before the day of observation. The average oldness level is the average value of the binary old music choices of the same calendar day in 2019.

The logistic regression maximises the following log-likelihood function:

3 $$I\left( \beta \right) = \mathop {\sum}\nolimits_i^N {\left[ {Y_i{\rm {ln}}\left( {p_i} \right) + \left( {1 - Y_i} \right){\rm {ln}}\left( {1 - p_i} \right)} \right]}$$

This work examines the following hypotheses:

(i) H1: *α*_2_ ≠ 0. The lockdown did not induce a sudden incremental change in old music listening behaviour. If *α*_2_ > 0, the lockdown induced a sudden incremental increase in old music listening.
(ii) H2: *β*_3_ = 0. The lockdown did not induce a change in the behavioural trend of old music listening. If *β*_3_ > 0, the lockdown induced a gradual increase in old music listening.
(iii) H3: *β*_1_ + *β*_3_ = 0. The behavioural tendency to listen to old music did not show any upward or downward trend during the lockdown. If *β*_1_+*β*_3_ > 0, listening to old music followed an upward trend after the lockdown.

## Results

**Fig. 4:**
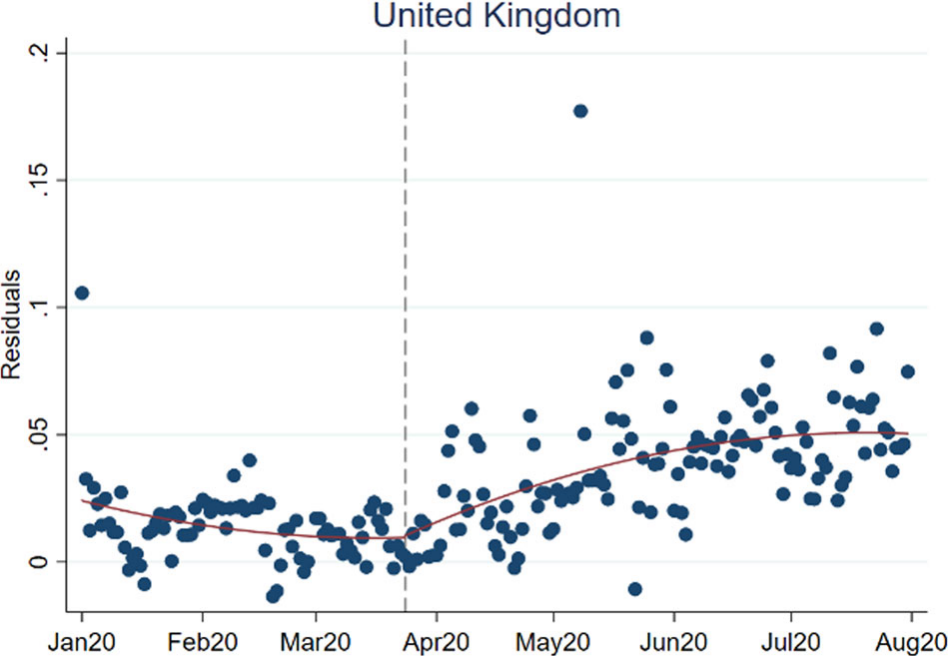
Bin-scatter plots of regression residuals, January–August 2020. *Notes*: Quadratic trends are shown in red curves. The behavioural tendency to listen to old music followed a downward trend before the lockdown was imposed, which is indicated by the dashed vertical line. The preference took an upward turn after the first lockdown day and gradually went up over time. *Source:* Author’s analysis and illustration of Spotify data.

The regression results are presented in Table 1. As shown in column 1, the trend in the preference for old music took a clearly positive turn (*β*_3_ > 0) and the subsequent trend went upward (*β*_1_ + *β*_3_ > 0). To illustrate the change in preference, Fig. 4 shows a bin-scatter graph that plots the residuals of a logistic regression that is identical to the baseline but excludes the time dimension and the lockdown binary indicator. In other words, the residual plot illustrates what the time component has explained in the baseline regression.^9^ It is obvious that the preference for nostalgic music took an upward turn just after the lockdown was imposed. Meanwhile, the lockdown effect is gradual as shown by the insignificant *α*_2_. On top of the lockdown effect, Covid-19 infections are also associated with more frequent listening to old music.

**Table 1:** Logistic regression of nostalgic music choice.

	(1) UK
*Pre-lockdown*	
Time trend (*β*_1_)	−0.0123 (0.0081)
Time trend-squared (*β*_2_)	−0.0001 (0.0001)
*During lockdown*	
Time trend (*β*_3_)	0.0174*** (0.0078)
Time trend-squared (*β*_*4*_)	0.0001 (0.0001)
*β*_1_ + *β*_3_	0.0064* (0.0823)
*β*_2_ + *β*_4_	0.0000 (0.9167)
Lockdown (*α*_2_)	0.0043 (0.1113)
Covid-19 incidence	0.2305*** (0.0538)
Total plays	0.5133 (0.5130)
New tracks	−0.0176*** (0.0033)
Average daily oldness level 2019	2.5536** (1.2361)
Weekday fixed effects	Yes
Number of obs.	4,166,040,525
Pseudo-*R*-squared	0.0263

Robust standard errors are in parentheses.

*Significant at 10%; **significant at 5%; ***significant at 1%.

**Fig. 5:**
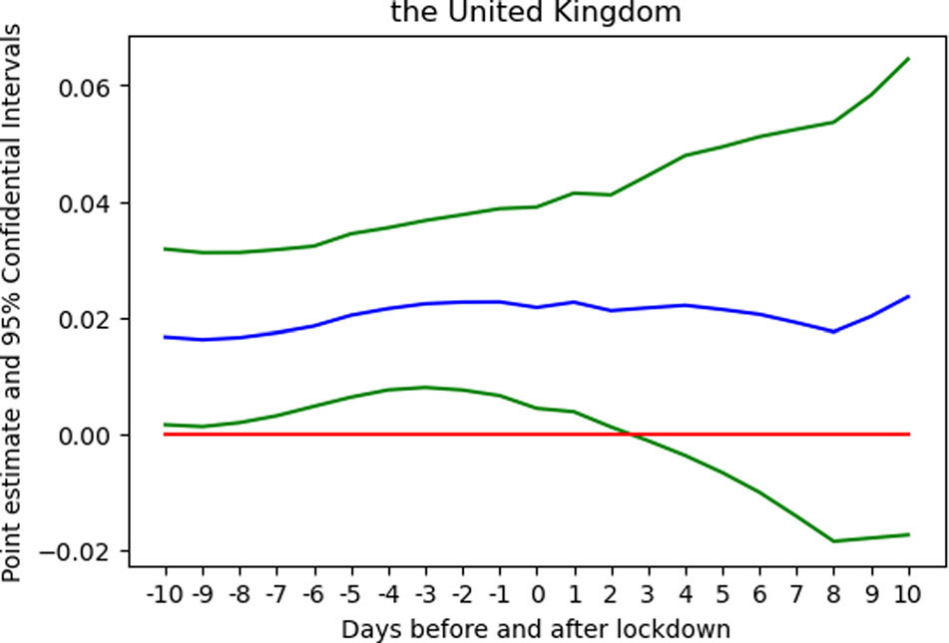
Estimated coefficients of the change in trend at 21 supposed break points. *Notes:* The blue line plots 21 estimates of the coefficient of the change in preference at a supposed breakpoint, while the green lines refer to the corresponding confidence intervals. The blue line is hump-shaped with the peak, roughly around *t* = 0. It shows that the change in preference was most marked at around the actual breakpoint (to be precise at *t* = −1). This result suggests that the lockdown sparked off an upward change in the preference for nostalgic music. *Source:* Author’s analysis and illustration of Spotify data.

Despite the finding, one may question whether the change in behaviour might not be due to the lockdown but to some other unknown factors. By repeating the same regression but supposing the lockdown began on another day (within the range of 10 days before and 10 days after the first lockdown day), I obtain 20 additional estimates of the change in preference (*β*_3_). Figure 5 plots the coefficients against the 21 dates. A point estimate refers to the magnitude of the change in slope/trend. A positive (negative) value means that the trend turns upward (downward). The larger the absolute value, the larger is the change in slope. The day where a peak (trough) is found corresponds to the starkest change in slope—a structural break.^10^ In Fig. 5, the peak is found on the day before the first lockdown day, suggesting that the lockdown indeed induced a structural break in the behaviour of old music listening.

**Fig. 6:**
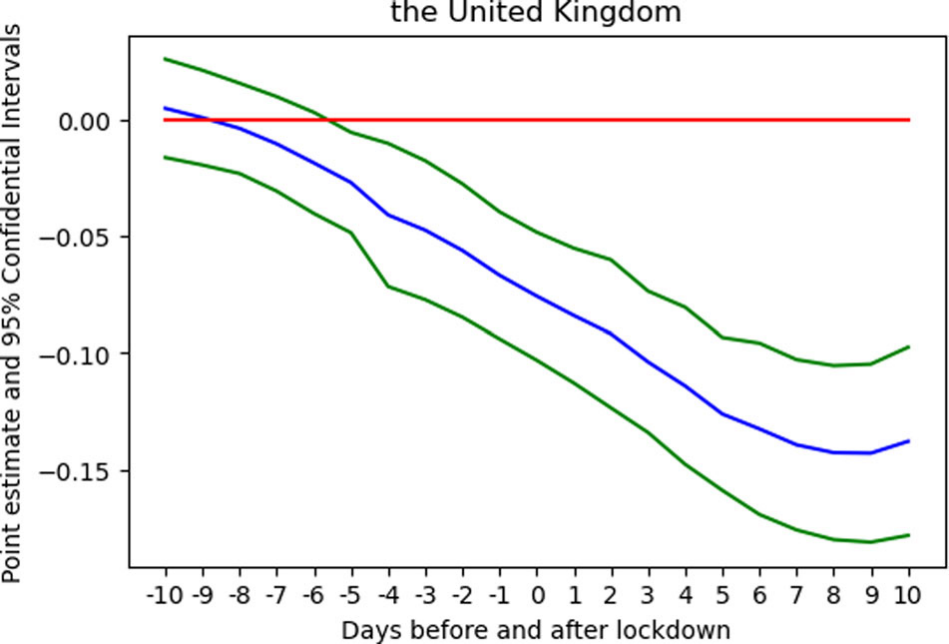
Estimated coefficients of the change in trend at 21 break points in 2019. *Note:* There was no sharp change in preference around *t* = 0. *Source:* Author’s analysis and illustration of Spotify data.

To investigate whether the change in preference could just be an annual pattern, another check moves the time frame to 2019 and supposes a lockdown was imposed on the same day in 2019 or any day within the range of 10 days before and 10 days after. Figure 6 reports the result. The curves are different from their counterparts in 2020; no hump-shaped curve is found. A large part of the curve is located in the negative zone. The curve is decreasing, i.e. music preference tends to shift towards recent songs and reaches a trough at 9 days after *t* = 0, suggesting that no upward, sharp change in the trend is present during the same period in 2019. No similar pattern is found in 2019, implying that the change in 2020 is not an annual phenomenon.

In short, data show that the lockdown very likely triggered an upward change in the trend of old music listening. To explain this phenomenon, this analysis resorts to the simplest explanation. People were shocked or saddened by the lockdown and sought emotion regulation strategies. Listening to old music helped them enjoy the positive effects induced by nostalgia.

## Which songs are back?

The previous section provides evidence of a shift in music preference towards nostalgic songs. Yet, merely being released in the past does not constitute the utility enjoyed by the listeners. This second part of the paper attempts to study which types of old songs came back onto people’s playlists during the lockdown.

In particular, existing research on music during Covid-19 has suggested a positivity bias (Hansen, 2022; Hansen et al., 2021). A survey found that respondents listened more to new and happy songs, as well as to music from their childhood (Ferreri et al., 2021). The present study aims to leverage Spotify streaming data and respond to this line of research. It is important to see if the nostalgic preference is indeed only a side effect of the potential positivity bias.

**Fig. 7:**
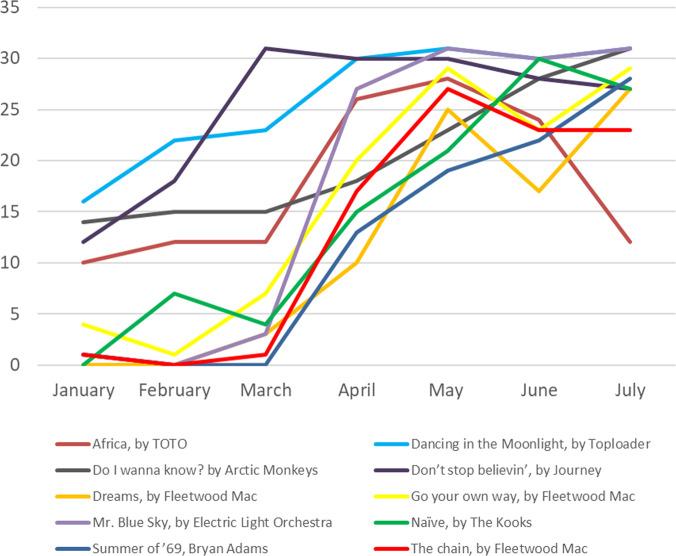
Number of times 10 selected revived songs hit the UK top 200, January–July 2020. *Note*: Only 10 of 28 are shown in this graph. *Source:* Author’s illustration of Spotify data.

To understand the change, it is helpful to single out the more prominent old songs that experienced a renaissance. To keep things transparent and tractable, I adopt the following selection process to identify these revivals.^11^ First, only those old songs that made it on the top 200 list at least a day each month from April to July 2020 are considered. Given the first condition, I define revivals as those in which the percentage of days hitting the top 200 in April–July 2020 is higher than in January–March 2020. Twenty-eight songs are identified from 4133 unique songs that hit the top 200 for at least a day during the 7-month period. Information on the 28 revivals is given in Table 2 and the performance of a selected 10 of them is visualised in Fig. 7.

**Table 2:** List and details of the 28 revived songs.

Title	Artist	Release date	Number of days hitting the top 200 (Jan.–Mar. 2020, 91 days)	Number of days hitting the top 200 (Apr.–Jul. 2020, 122 days)
‘(Sittin’ on) The Dock of the Bay’	Otis Redding	8 January 1968	0	13
‘Africa’	Toto	8 April 1982	34	90
‘All of Me’	John Legend	30 August 2013	21	37
‘Brown Eyed Girl’	Van Morrison	June 1967	0	14
‘Budapest’	George Ezra	27 June 2014	1	54
‘Can’t Hold Us’ (featuring Ray Dalton)	Macklemore & Ryan Lewis	9 October 2012	22	118
‘Chasing Cars’	Snow Patrol	6 June 2006	7	52
‘Dancing in the Moonlight’	Toploader	August 1970	61	122
‘Do I Wanna Know?’	Arctic Monkeys	1 January 2013	44	100
‘Don’t Look Back in Anger’ (Remastered)	Oasis	2 October 1995	71	109
‘Don’t Stop Believin”	Journey	October 1981	61	115
‘Don’t Stop Me Now’ (2011 Mix)	Queen	10 October 1978	39	88
‘Dreams’	Fleetwood Mac	4 February 1977	3	79
‘Go Your Own Way’	Fleetwood Mac	4 February 1977	12	101
‘Here Comes the Sun’	The Beatles	26 September 1969	1	49
‘Hips Don’t Lie’ (featuring Wyclef Jean)	Shakria, Wyclef Jean	28 November 2005	0	21
‘I Wanna Dance with Somebody’ (Who Loves Me)	Whitney Houston	2 June 1987	16	74
‘Let Her Go’	Passenger	24 July 2012	3	51
‘Lovely Day’	Bill Withers	29 October 1977	0	29
‘Mr Blue Sky’	Electric Light Orchestra	28 January 1978	4	119
‘Naive’	The Kooks	27 March 2006	11	93
‘Rocket Man (I Think It’s Going to Be a Long, Long Time)’	Elton John	19 May 1972	13	21
‘September’	Earth, Wind & Fire	11 September 2013	5	27
‘Summer of ‘69’	Bryan Adams	17 June 1985	14	69
‘The Chain’	Fleetwood Mac	4 February 1977	2	90
‘Wake Me Up’	Avicii	13 September 2013	7	69
‘Waves’ (Robin Schulz Radio Edit)	Mr Probz	7 February 2014	0	28
‘You Can Call Me Al’	Paul Simon	12 August 1986	0	10

*Note*: The selection is based on comparing the percentages of hitting the top 200 during the two periods.

*Source:* Author’s computation using Spotify data.

A common trend is identified in Fig. 7. These songs were played more frequently from March and reached their peaks in May before a gradual downward adjustment towards July. Studying their lyrics, I find some positive, joyful and upbeat songs, such as ‘Mr Blue Sky’ by Electric Light Orchestra:

‘Hey there Mr Blue. We’re so pleased to be with you. Look around see what you do Everybody smiles at you.’

The same applies to ‘Dancing in the Moonlight’ by Toploader:

‘Dancin’ in the moonlight. Everybody’s feelin’ warm and bright. It’s such a fine and natural sight. Everybody’s dancin’ in the moonlight.’

This finding is interesting and consistent with the existing literature that people became more inclined to listen to positive songs during the pandemic, pondering a further question. Given people’s tendency to listen to positive songs during the pandemic, they were more likely to find their preferable music from the past since the set of old songs is much bigger than the set of recent songs. The revival of old songs could only be a side effect of searching for positivity. The question is whether the revival of old music occurred for both positive and negative songs.

To address it, I expand the scope from the 28 revived old songs to all old songs in the sample. That enables their comparison with all recent songs in terms of seven audio feature metrics (acousticness, danceability, energy, liveness, loudness, tempo and valence) compiled by Spotify.^12^

The sample contains in total 4133 songs, in which 44 songs are at the borderline of passing from being recent to being old during the sample period. Removing the borderline cases leaves 3221 recent songs and 868 old songs in the sample. Table 3 reports the mean of each sample and the *p*-value of an unequal-variance *t*-test for each of the seven dimensions. The statistics show that the two samples are significantly different in all but one dimension. On average, old songs are more acoustic, energetic and lively while being less loud and less danceable, and most importantly, they are more positive as shown by the valence measure.

**Table 3:** Summary statistics of audio features of nostalgic and recent songs.

Dimension	Acousticsness	Danceability	Energy	Liveness	Loudness	Tempo	Valence
Mean (old) *N* = 868	0.2554	0.6043	0.6535	0.1904	−7.4438	119.9292	0.5829
Mean (recent) *N* = 3221	0.2346	0.6663	0.6302	0.1751	−6.73	120.8187	0.4657
*p*-value (2-tail)	0.0607*	0.0000***	0.0044***	0.0084***	0.0000***	0.3960	0.0000***

Source: Author’s computation using Spotify data.

***Significant at the 1% level; **Significant at the 5% level; *Significant at the 10% level.

## Audio feature analysis

The simple statistical analysis finds that old songs, which successfully charted the top 200 at least a day during the sample period, are on average associated with a higher value of valence. Valence is of special interest as it gives a general score for a song’s positivity. The higher the score, the happier, more joyful and more euphoric is the song. This finding complicates the original research question since the return of old music, even if it was triggered by the lockdown, could merely be a by-product of a search for positivity.

To further investigate, I classify each song into one of five categories, namely, positive old, negative old, positive recent, negative recent songs and the rest. While following the same dichotomic definition of old music, I classify songs into positive (valence score > 0.667), negative (valence score < 0.333) and neither. Interestingly, these arbitrary thresholds almost match the 75-percentile (0.662) and the 25-percentile (0.317).

**Fig. 8:**
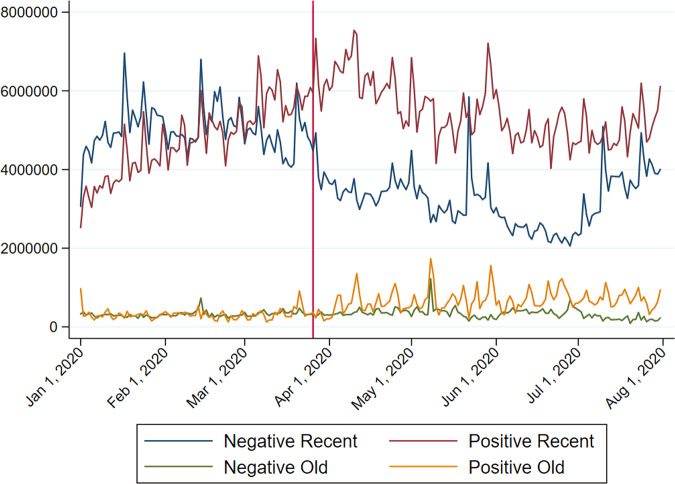
Changes in the total number of plays of four types of songs. *Source:* Author’s illustration of Spotify data.

Next, I aggregate songs of the same category and plot the sum of the number of plays against time in Fig. 8. Before the lockdown set in, as indicated by the vertical red line, positive and negative songs were not distinguishable in terms of their number of plays. Their difference becomes discernible during the lockdown period, with positive songs consistently outperforming negative ones for both old and recent songs. While positive old songs followed an upward trend from 26 March 2020, recent songs, both positive and negative, endured a downward trend.

**Fig. 9:**
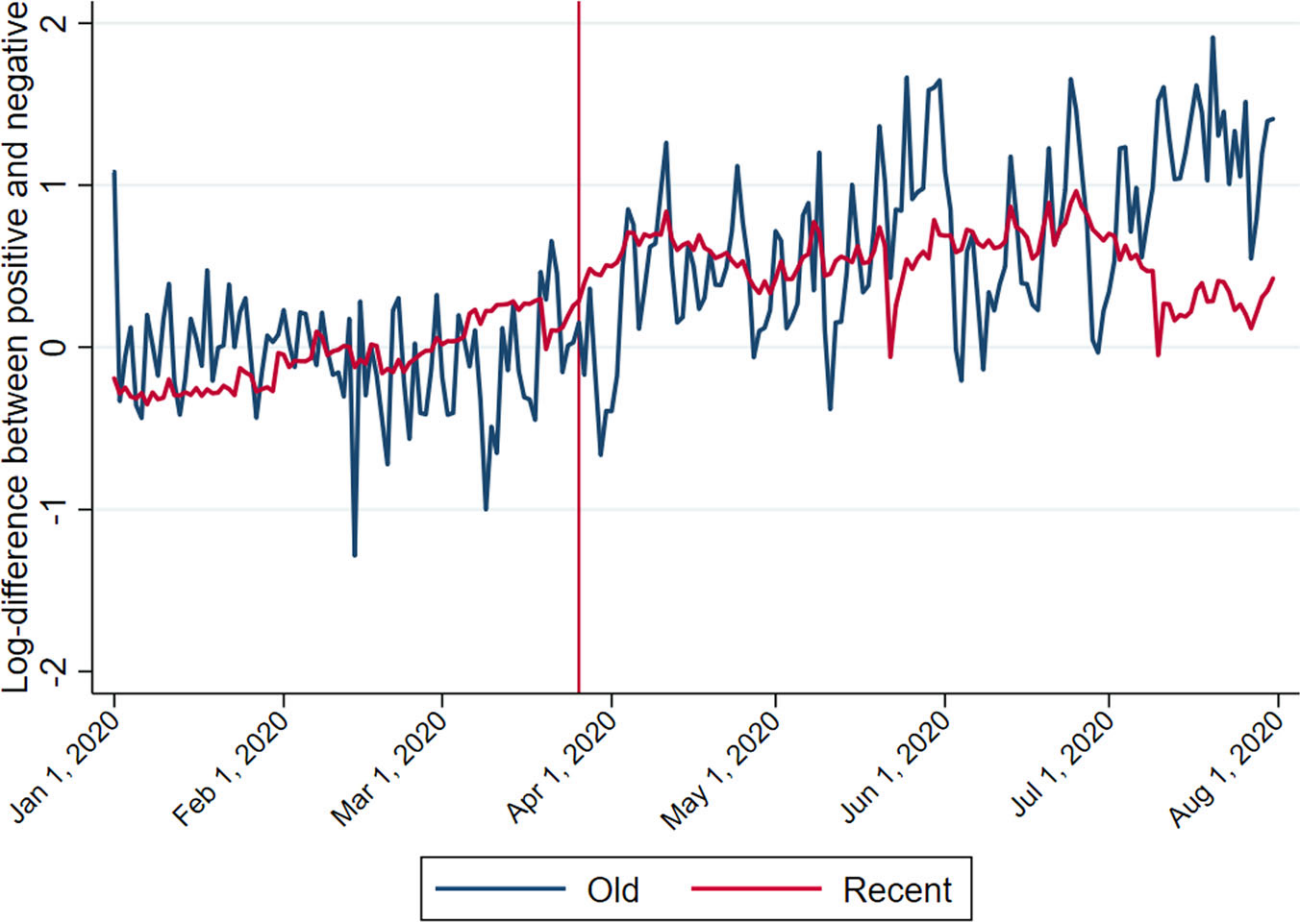
Log difference in the total number of plays between positive and negative songs. *Source:* Author’s illustration of Spotify data.

**Fig. 10:**
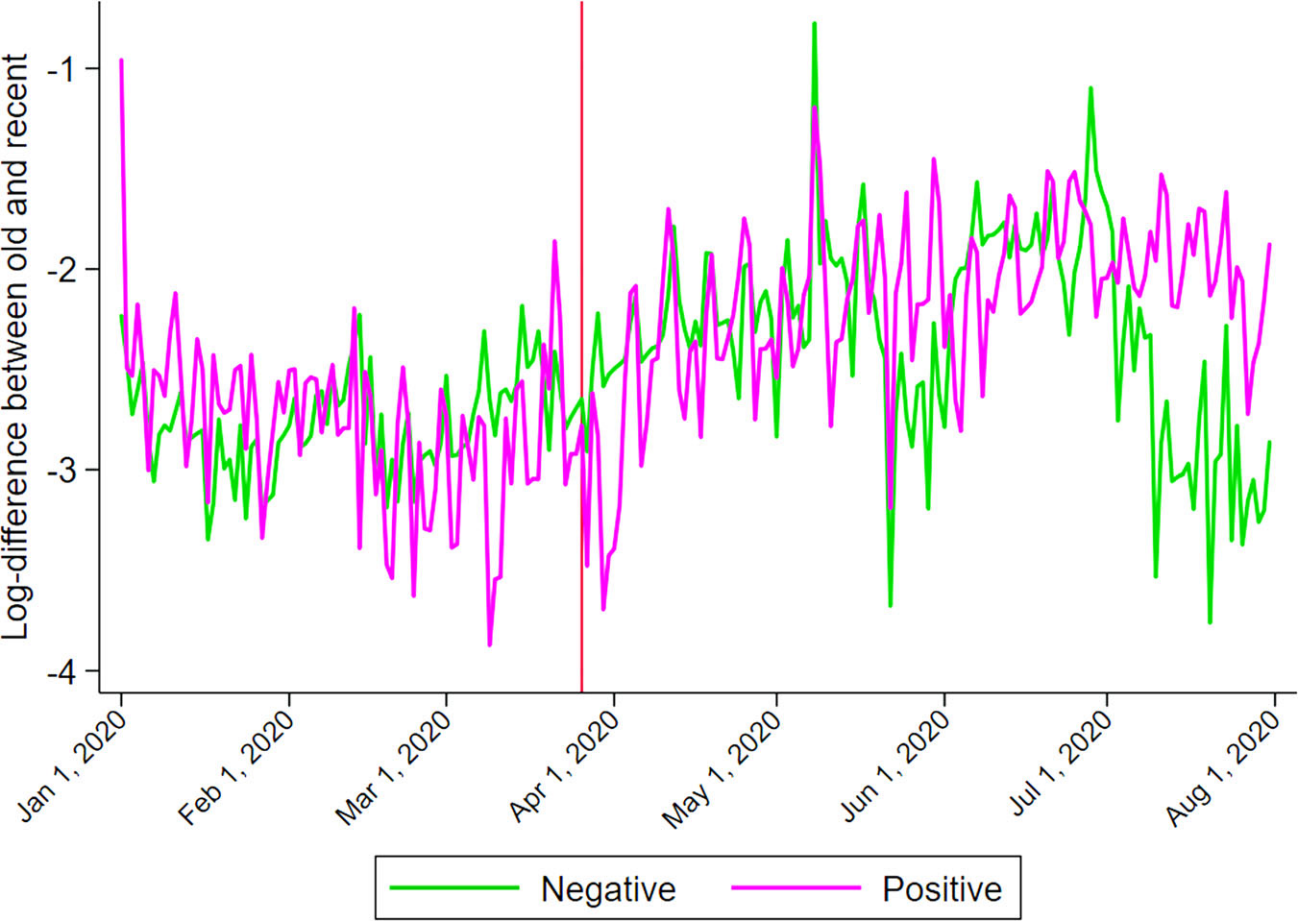
Log difference in the total number of plays between old and recent songs. *Source:* Author’s illustration of Spotify data.

The analysis moves on to determine, first, if the lockdown triggered a preference shift towards positive songs, and second if the rise in listening to old music is not due to the rise in positive music listening. I construct four dependent variables, namely, the log differences in the total number of plays between positive and negative songs of either an old or recent type and the log differences in the total number of plays between old and recent songs of either a positive or negative type. Figures 9 and 10 illustrate the changes over time of these four variables. As hinted at in Fig. 8 and clearly shown in Fig. 9, the preference for positive songs over negative ones is more pronounced following the lockdown: most of the values are above zero after the lockdown began. But that could be a continuation of their respective trends. The divergence between old and recent songs shown in Fig. 10 is similar, as both of them followed an upward trend, though short-lived for negative songs after the lockdown.

Graphical investigation does not provide a clear answer. Next, I conduct a time series regression analysis for the four variables to check if the lockdown altered their respective trends. Following the standard steps for conducting a time series analysis, I first check for stationarity using an augmented Dickey–Fuller test and ensure that the time series is stationary by filtering out the effects of total daily plays and day-of-the-week fixed effects. Next, I determine the order of lags by comparing the Bayesian information criterion (BIC).^13^ Suppose the time series is an AR(1) process. To test if the lockdown altered music preference, I model the preference as follows:

4 $$y_t = \mu _1 + \mu _2{\rm {Lockdown}}_t + \mu _3t + \mu _4{\rm {Lockdown}}_tt + \mu _5y_{t - 1} + \varepsilon _t$$

The quadratic trend component is removed because the inclusion of the lagged value of the dependent variable has very much captured the quadratic trend component. The log of the 7-day moving average of the number of Covid-19 cases is also dropped so as to avoid a serially correlated regressor in a time series analysis.

The coefficients of interest are *μ*_2_ and *μ*_4_. The former captures any change in the intercept term (the fixed component), while the latter shows if there is any change in the slope (the time-dependent component). A positive and significant *μ*_2_ implies that the lockdown caused Spotify users in the UK to listen to a certain type of music (e.g. more positive music in the case of the log difference in the total number of plays between positive and negative songs being the dependent variable) and the effect is persistent throughout the lockdown period. A positive and significant *μ*_4_ indicates a change in the linear trend component, implying that the lockdown gradually caused Spotify users to listen to a certain type of music. I report the results of the four regressions in Table 4.

**Table 4:** Time series analysis of the preference for nostalgia/positivity.

	(1)	(2)	(3)	(4)
Sample	Positive	Negative	Recent	Old
Dependent variable	Old–Recent		Positive–Negative	
Lockdown (*μ*_2_)	0.1449* (0.0809)	0.1706** (0.0657)	0.1005*** (0.0372)	0.1252 (0.0817)
Pre-lockdown Time trend (*μ*_3_)	−0.0028** (0.0012)	−0.0006 (0.0007)	0.0017*** (0.0006)	−0.0005 (0.0011)
Post-lockdown Time trend (*μ*_4_)	0.0060*** (0.0015)	−0.0006 (0.0010)	−0.0020*** (0.0007)	0.0041*** (0.0014)
First lag (*μ*_5_)	0.5124*** (0.0714)	0.4172*** (0.0746)	0.5971*** (0.0615)	0.4488*** (0.0761)
Second lag				0.1008 (0.0675)
Number of lags	1	1	1	2
*R*-squared	0.6249	0.2402	0.7381	0.6813
Number of obs	212	212	212	211

Robust standard errors are in parentheses.

*Significant at 10%; **significant at 5%; ***significant at 1%.

Notably, each dependent variable presents some degree of autocorrelation. In the sample of positive songs, as shown in column (1), we observe an incremental lockdown effect in listening to old music and a subsequent gradual rise after the lockdown. Column (2) shows the result for the sample of negative songs, where we also find a persistent lockdown effect but no further subsequent rise. Note that the two results do not necessarily mirror one another. In the sample of recent songs, we likewise observe an incremental increase but the effect is short-lived; a gradual reversal follows, as shown by the significant and negative post-lockdown trend adjustment. In the sample of old songs, there is no significant incremental increase, but a gradual subsequent rise appears after the lockdown. The time series regression analysis largely presents the same results as the graphical illustrations.

The results point to some interesting findings. First, the case of more frequent nostalgic music listening during the lockdown is well established, and the effect is more long-lasting in the sample of positive songs. Probably reflecting the same fact but from another angle, the rise in positive music listening persists longer if the songs are old. Second, in the sample of recent songs, music listeners initially prefer positivity but the effect wanes over time, while the average positivity level of the sample of old songs gradually increases without a significant reversal during the sample period. In short, the findings support the hypothesis that people turned to nostalgic music during the lockdown. Moreover, nostalgia-seeking behaviour is closely associated with a preference for positivity.

Music choices are personal decisions. The findings do not suggest that listening to old and happy songs will lead to positive impacts for everyone, but they show that most Spotify users responded to changes in the outside world by shifting their music preference towards old, very likely nostalgic, and positive songs.

## Discussion

As mentioned above, this work explores music streaming data collected from Spotify, which does not allow investigating the emotions experienced by music listeners. Yet, the evidence presented stimulate interesting discussions on both the explanations of the identified phenomena and some implications for research on music and psychology.

### What does nostalgic music provide?

This work suggests that the lockdown caused Spotify users in the UK to seek pleasure from the music of their past. Why nostalgic music? What do the songs of one’s past provide? It is certainly not freshness, which is mainly found in new releases. The pandemic, or more precisely the first lockdown, induced widespread fear and distress in the population, which led people to seek nostalgic music. It was not just an isolated phenomenon but systematically observed in the nation.

In other words, nostalgic music seems to provide a healing effect for people in difficult times. The songs of one’s past that remain in the choice set must be considered ‘good’ by the person to a certain extent. They are likely to be associated with some pleasant feelings or memories. Research based on a survey of 570 participants also showed that nostalgic music listening as an emotion regulation strategy has a positive impact on well-being (Gibbs and Egermann, 2021). In fact, music therapy has long been an established discipline (William et al., 2008) and its healing effect has also been explored during the pandemic time (Martinez-Castilla et al., 2021; Vidas et al., 2021). While nostalgic music as an emotion regulation strategy is less understood, the healing effect of nostalgic music for dementia patients has been extensively studied (Koger et al., 1999; Vink et al., 2003).^14^

The exact mechanism leading from nostalgia to emotion regulation is a promising research direction. One of the reasons is that the virtual space nostalgic music creates gives the listener a route to escape from an uncertain and stressful reality. Nostalgic music is by definition some music one knows well, connected to one’s past. It gives a sense of certainty as the listener is familiar with the melody and thus provides quality assurance. It also reminds one amid the lockdown of the days associated with nostalgic music when people were free to interact.

This work does not study music listeners’ psychology or emotions, as such information is not available in the music streaming data, but provides evidence of human behavioural changes triggered by a Covid-19 lockdown. Together with existing findings in the recent but fast-growing literature, this work hints at the fact that nostalgic music listening is an effective emotion-regulation strategy in times of difficulty.

### Nostalgia consumption

The literature provides abundant evidence of the impacts of Covid-19 on mental health (Sun et al., 2020; Pfefferbaum and North, 2020; Mucci et al., 2020). The psychological effects of the pandemic may easily translate into changes in behaviour as rational individuals seek remedies to counter any psychological distress. Nostalgia consumption during the pandemic has been discussed in recent research (Gammon and Ramshaw, 2021; Weed, 2020).

Nostalgia has also long been a research topic in consumer studies (Holbrook, 1993; Holbrook and Schindler, 2003; Holak et al., 2007). Marketing strategies based on nostalgic feelings have been widely adopted (Unger et al., 1991; Russell, 2008). Hirsch (1992) suggested that by defining nostalgia as a yearning for an idealised past, nostalgia marketing induces the displacement of idealised past emotions onto objects. Difficult times are thus successful ones for nostalgia marketing alluding to a better past (Spaid, 2013).

The pandemic has altered people’s consumption patterns (Hall et al., 2020) and heavily affected the consumption of cultural goods. Weed (2020) discussed how the cancellation of sports events led TV channels to replay matches from the past, and the potentially restorative nature of this form of nostalgia in supporting well-being during the lockdown.

### Sad songs and happy songs

Saarikallio and Erkkilä (2007) identified two main roles of music in mood regulation: *mood improvement* and *mood control*. Mood improvement relies heavily on putting oneself in a desirable positive mood, but could also be achieved by listening to sad music that helps the listener to gain understanding of a sorrowful experience (Larsen, 2009). Mood control is an attempt to take control of one’s emotions, to calm down, to make sense of one’s thoughts and feelings. Any type of music could perform this role, reflecting one’s personal and contextual needs. In fact, sad songs are sometimes preferred by people who are feeling sad (Van den Tol and Edwards, 2011). Using experimental data, Friedman et al. (2012) showed that sad people tend to avoid listening to happy songs because they feel it is inappropriate.

The concern of inappropriateness may be misplaced in the context of Covid-19 because most people under the first lockdown did not suffer from the virus or have a relative or friend died because of it. Everyone perceives the pandemic or lockdown differently. While some were saddened or frightened, others may have just felt bored. Users, depending on their moods and preferences, may have resorted to either positive or negative songs.

Music listening during the pandemic showed a positivity bias (Ferreri et al., 2021; Hansen, 2022; Howlin and Hansen, 2022), suggesting that positive songs seemed to have been a more effective tool in combating the negativity caused by the pandemic. This work finds a similar but subtler pattern: the upsurge of positive music listening was more persistent if it was nostalgic.

## Conclusion

This study first provides evidence that the UK’s lockdown in late March 2020 triggered Spotify users in the UK to listen more frequently to songs released at least 5 years ago. The phenomenon is very likely associated with nostalgia-seeking behaviour during a lockdown when people were worried, saddened or stressed by the uncertainties ahead.

The second part of the study attempts to determine whether the ‘revival’ of old songs was merely a consequence of an increase in the preference for positive songs. The analysis shows that nostalgia-seeking behaviour is present across the samples of positive and negative songs, supporting the hypothesis that nostalgic music listening is to a certain extent independent of the positivity bias of music listening behaviour during the pandemic found in the literature. Yet, this work also finds evidence that nostalgia-seeking behaviour and a preference for positive songs reinforced each other during the lockdown, as the surge in positive old music was more persistent than that in positive recent music.

## Supplementary information

Appendix

## Data Availability

The datasets generated during and analysed during the current study are not publicly available because Spotify owns the right to distribution of the data but are available from the author on reasonable request.

## Footnotes

1 Yeung (2020) has shown that lockdown triggered a gradual rise in nostalgic music listening in all six countries on Spotify. I focus on the UK mainly because my limited understanding of other languages does not allow me to perform a deep-dive analysis of the lyrics of, and thus the general messages conveyed by, the songs.

2 See https://www.legislation.gov.uk/uksi/2020/350/pdfs/uksi_20200350_en.pdf

3 See https://data.europa.eu/data/datasets/covid-19-coronavirus-data?locale=en

4 See https://kommandotech.com/statistics/spotify-user-statistics/

5 This explanation would see a long-lasting effect but the results here show that the upward trend in behaviour of listening to old songs reversed towards the relaxation of confinement measures.

6 The lockdown was announced on 23 March 2020.

7 The information is clustered in songs on a day, rather than plays of songs. Each entry contains information about the song and the frequency of plays during the day. Therefore, there are 42,400 entries (200 songs in the chart times 212 days) but 4.166 trillion plays of songs. To perform the logistic regression analysis, each entry (song during a day) is weighted by its frequency of plays. In other words, the weight is the number of duplicates of the song in a day. A logistic regression analysis without specifying the weights would make less sense, as it would consider each charted song to be equivalent in popularity.

8 The baseline regression includes the daily average oldness level of 2019, which should sufficiently address this concern.

9 The bin-scatter plot groups the *x*-axis variable into equal-sized bins computes the mean of the *x*-axis and *y*-axis variables within each bin, and then creates a scatterplot of these data points.

10 No structural break is identified if no peak or trough is present.

11 Calling them ‘revivals’ may be exaggerating because some of these songs were frequently played before the pandemic struck. Readers are asked to bear with the term.

12 See https://developer.spotify.com/documentation/web-api/reference/#/operations/get-several-audio-features for more detailed descriptions of the audio features.

13 The result of using the Akaike information criterion (AIC) for the lag order selection is provided in the Appendix.

14 While a Google Scholar search of ‘music’ + ‘emotion regulation’ gives about 35,200 results, a search of ‘nostalgic music’ + ‘emotion regulation’ gives only 57, among which many are papers written during the current pandemic. Data were retrieved on 26 March 2023.
