# Supplementary material for: Revival of positive nostalgic music during the first Covid-19 lockdown in the UK: evidence from Spotify streaming data
Source: Humanit Soc Sci Commun. 2023 Mar 29;10(1):132. doi: 10.1057/s41599-023-01614-0 (PMC10050806; doi:10.1057/s41599-023-01614-0)
Supplement: Supplementary file 1 — Appendix [file 41599_2023_1614_MOESM1_ESM.docx]

# Appendix

## Preparation of the time series

### Step 1. Ensure stationarity

The total number of daily plays, which is autocorrelated, and day-of-the-week fixed effects have been filtered out from the four time series. I then conduct an augmented Dickey–Fuller unit-root test and present the results in Table A1.

Table A1: Augmented Dickey–Fuller test

|  | **(1)** | **(2)** | **(3)** | **(4)** |
| --- | --- | --- | --- | --- |
| Sample | Positive | Negative | Recent | Old |
| Dependent variable | Old–Recent | | Positive–Negative | |
| Z(t) | -5.618 | -8.982 | -5.183 | -5.130 |
| MacKinnon approximate p-value | 0.000 | 0.000 | 0.000 | 0.000 |
| Number of obs | 212 | 212 | 212 | 212 |

*Notes*: The null hypothesis is the time series and is non-stationary. The test result rejects the null hypothesis.

### Step 2. Optimal lag-order selection

As the augmented Dickey–Fuller test rejects the time series being non-stationary (i.e. unit-root), I proceed to search for the optimal order of lags in the autoregressive regression as shown in Table A2.

Table A2: Optimal lag-order selection by the AIC and BIC

|  | **(1)** | **(2)** | **(3)** | **(4)** | **(5)** | **(6)** | **(7)** | **(8)** |
| --- | --- | --- | --- | --- | --- | --- | --- | --- |
| Sample | Positive | | Negative | | Recent | | Old | |
| Dependent variable | Old–Recent | | | | Positive–Negative | | | |
| Criterion | AIC | BIC | AIC | BIC | AIC | BIC | AIC | BIC |
| Optimal lag order | 4 | 1 | 4 | 1 | 5 | 1 | 4 | 1 |

*Note:* AIC refers to the Akaike information criterion. BIC refers to the Bayesian information criterion.

### Step 3. Autoregressive regression

In the main text I present the results of the autoregressive regressions with the lag order suggested by the BIC. I show the results with the lag order suggested by the AIC in Table A3.

Table A3: Autoregressive regression with the lag order suggested by the AIC

|  | **(1)** | **(2)** | **(3)** | **(4)** |
| --- | --- | --- | --- | --- |
| Sample | Positive | Negative | Recent | Old |
| Dependent variable | Old–Recent | | Positive–Negative | |
| Lockdown ($\mu_{2}$) | 0.1576*  (0.0853) | 0.1471**  (0.0639) | 0.1068***  (0.0383) | 0.1338  (0.0882) |
| Pre-lockdown  Time trend ($\mu_{3}$) | -0.0034***  (0.0013) | -0.0011*  (0.0006) | 0.0013**  (0.0006) | -0.0009  (0.0013) |
| Post-lockdown  Time trend ($\mu_{4}$) | 0.0064***  (0.0017) | 0.0001  (0.0010) | -0.0016**  (0.0007) | 0.0045***  (0.0017) |
| Number of Lags | 4 | 4 | 5 | 4 |
| R-squared | 0.6388 | 0.2704 | 0.7095 | 0.6825 |
| Number of obs | 209 | 209 | 208 | 208 |

Using a different lag order does not change the findings presented in the main text.
